# P-797. Household Contact Tuberculosis Screening Experience and Predictors of Tuberculosis Disease Diagnosis in Rural Tanzania

**DOI:** 10.1093/ofid/ofae631.989

**Published:** 2025-01-29

**Authors:** Ghassan ILAIWY, Saning’o Lukumay, Domitila Agustino, Paulo Mejan, Kusulla Simeon, Estomih Mduma, Scott Heysell, Tania Thomas

**Affiliations:** University of Virginia, Charlottesville, Virginia; Haydom Lutheran Hospital, Haydom, Manyara, Tanzania; Haydom Lutheran Hospital, Haydom, Manyara, Tanzania; Haydom Lutheran Hospital, Haydom, Manyara, Tanzania; Haydom Lutheran Hospital, Haydom, Manyara, Tanzania; Haydom Lutheran Hospital, Haydom, Manyara, Tanzania; University of Virginia, Charlottesville, Virginia; University of Virginia, Charlottesville, Virginia

## Abstract

**Background:**

One third of people with TB disease (PWTB) remain undiagnosed, experiencing prolonged illnesses before diagnosis. To close this gap, the World Health Organization and national guidelines recommend TB screening for all household contacts (HHCs) of index PWTB. HHC TB screening experiences vary between countries, and between rural and urban parts within the same country. Therefore, we aimed to characterize the HHC TB screening and explore predictors of TB disease diagnosis among HHCs in Rural Tanzania.

**Methods:**

We used data from a prospectively enrolled cohort of index PWTB and their HHCs in Haydom, Tanzania. We describe the testing recommended as part of HHC TB screening and use a multivariate regression model to explore predictors of TB disease diagnosis.

**Results:**

The cohort enrolled 120 index PWTB with 398 HHCs. 261 HHCs (66%) from 85 households completed TB screening with sputum and chest x-ray recommended for 121 (46%) and 3 (1%) HHCs respectively. 18 HHCs (4.5%) were diagnosed with TB disease, 14 of them with no sputum or CXR recommended. HHCs with TB symptoms and HHCs of index PWTB with pulmonary TB or HIV were more likely to be diagnosed with TB. Only 10 HHCs completed six-months of daily isoniazid TPT out of 61 HHCs who started TPT (16.3%) and 79 HHCs who were eligible (12.7%).

**Conclusion:**

The recommended sputum and imaging studies to complete HHC TB screening and short TPT regimens have suboptimal penetration in this rural high-burden setting. Innovative testing and coverage modalities are needed to bridge the gap.

**Disclosures:**

**All Authors**: No reported disclosures

